# Emerging pharmacological targets in migraine therapy: CGRP and beyond

**DOI:** 10.3389/fphar.2026.1924544

**Published:** 2026-09-09

**Authors:** D. Atraszkiewicz, D. Dahiya

**Affiliations:** Leeds Teaching Hospitals NHS Trust, Leeds, United Kingdom

**Keywords:** 5-HT_1F_ receptor agonist, CGRP-calcitonin gene-related peptide, iNOS-inducible nitric oxide synthase, mGluR5 allosteric modulator, migraine, novel pharmacological approaches, PACAP (pituitary adenylate cyclase-activating polypeptide), potassium channel (K_ATP_)

## Abstract

Migraine is a prevalent, complex, and disabling neurological disorder. Available treatments consist of repurposed drugs, with medication switching and treatment failure a common occurrence. Pathophysiological models have shifted from migraine being a primarily vascular disorder to a complex network disease encompassing cortical, hypothalamic, thalamic, brainstem, trigeminovascular, and meningeal structures. These areas are interconnected by cascades of multiple signalling transmitters and hormones. This review explores how these molecules may offer novel pharmacological targets for efficacious treatments tailored to the pathophysiology of migraine. Calcitonin gene-related peptide (CGRP) directed therapies include antagonists (“gepants”) and monoclonal antibodies. Both drug classes have shown promising effects in managing pain in acute migraine attacks and in prophylactically reducing headache frequency, even within treatment-resistant migraine populations. Animal studies have shown drugs targeting pituitary adenylate cyclase-activating polypeptide (PACAP) signalling to reduce meningeal vasodilation and chronic allodynia. PACAP ligand-directed monoclonal antibodies offer therapeutic promise in patients with treatment-resistant migraine; however, this evidence base is limited and requires further research. Amylin may offer a novel therapeutic target for migraine; however, currently no such therapies have been developed. Neurokinin receptor antagonists have so far shown little promise in clinical trials, further challenging the role of substance p and neurogenic inflammation in migraine pathophysiology. Building upon the success of triptan therapy, novel serotonergic medications such as 5-HT_1F_ agonists (‘ditans’) have shown positive benefit in clinical studies as an alternative treatment to triptans. Additional emerging molecular targets include K_ir_6.1/SUR2B K_ATP_ and K_v_7 potassium channels, together with GluK1 and mGluR5 glutamate receptors. In contrast, despite a strong mechanistic rationale, therapeutic strategies targeting orexinergic pathways, nitric oxide synthase, and phosphodiesterase/cyclic nucleotide signalling have yet to demonstrate clinical efficacy in migraine. Overall, CGRP, PACAP, and serotonergic signalling offer the greatest promise as emerging pharmacological targets for migraine therapy. Most other emerging pathways remain in early, preclinical stages, with further research needed to bridge the gap between pathophysiological plausibility and robust clinical translation.

## 1 Background

Migraine is estimated to affect 14%–15% of people worldwide (Steiner and Stovner, 2023). The global disease burden of the disorder has increased significantly in recent years (Safiri et al., 2022). In the UK, £150 million/year is spent on migraine treatment and £4.4 billion is lost to the economy due to migraine-related work absence (NHS England, 2020).

The International Classification of Headache Disorders third edition (ICHD-3) defines chronic migraine (CM) as “headache occurring on 15 or more days/month for more than 3 months, which, on at least 8 days/month, has the features of migraine headache” (Headache Classification Committee of the International Headache Society IHS, 2018); however, this definition may soon be updated (Goadsby et al., 2025). Migraine diagnoses that do not meet this criteria are classified as “episodic migraine” (EM), of which there are multiple subtypes (Headache Classification Committee of the International Headache Society IHS, 2018). The mechanisms underpinning transformation to CM remain unclear (Atraszkiewicz, 2021); key risk factors include female sex (three times higher prevalence than men), low socioeconomic status, obesity, and psychiatric co-morbidities (Scher et al., 2008; Bigal and Lipton, 2006; Ashina et al., 2012; Buse et al., 2010).

Migraine is notoriously difficult to treat (Diamond et al., 2007). Patients are commonly prescribed progressive oral regimens of repurposed drugs, including: non-steroidal anti-inflammatory drugs (NSAIDs), triptans, beta-blockers, anti-epileptic medications, and neuropathic agents. Medication switching and re-initiation is very common, especially in CM (Hepp et al., 2017). Subsequently, despite having an estimated global prevalence of 1%–2% (Burch et al., 2019), CM has a high likelihood of severe headache impact (Buse et al., 2012) and accounts for the majority of migraine’s societal economic burden (Leonardi et al., 2019; Lanteri-Minet, 2014).

For patients whose symptoms persist despite multiple oral pharmacological treatments, regional procedures have gained increasing popularity in recent years. In a 2019 survey of British Association for the Study of Headache (BASH) members, 80% of respondents (48% response rate; mostly consultant neurologists, general practitioners, and nurses) performed peripheral nerve blocks for headache disorders—of which, greater occipital nerve blockade (GONB) was the most common (98%) (Idrovo et al., 2019). Despite this, there are no relevant UK guidelines for the procedure and a very limited evidence-base to advocate for its longer-term efficacy and use (Atraszkiewicz et al., 2025). Similarly, botulinum toxin type A (BoNT-A) injections show no therapeutic benefit over placebo in treating EM (Chamani et al., 2026). However, BoNT-A does significantly reduce headache frequency, headache severity, and medication use in CM (Chamani et al., 2026). Although efficacy was inferior to oral topiramate therapy, BoNT-A demonstrated improved tolerability with lower rates of treatment-related discontinuation (Chamani et al., 2026). Furthermore, BoNT-A injections may have a specific role in managing disability in intractable CM for patients with no pain-free time (Atraszkiewicz et al., 2022).

Non-pharmacologic approaches to migraine therapy also have variable efficacy findings. Pulsed radiofrequency neuromodulation has a low-quality evidence base for treating headache disorders (Oliveira et al., 2024). Though occipital nerve stimulation may offer promise in intractable CM (Alrahbeni et al., 2024), implantable nerve stimulators have a 31.5% rate of complications requiring return to the operating theatre (Ducic et al., 2014). Surgical nerve decompression may significantly improve migraine intensity (Alrahbeni et al., 2024) and has superior efficacy compared to radiofrequency approaches (Ducic et al., 2014) however, patient accessibility is limited and post-operative complications—such as paraesthesia—are experienced by almost one in three patients (ElHawary et al., 2022).

There is, therefore, a pertinent need for novel migraine treatments that are: 1) non-invasive; 2) tailored to migraine pathophysiology; 3) demonstrate clear efficacy in reducing long-term patient disability; and 4) are readily accessible within primary care settings. This paper explores the emerging pharmacological targets for such interventions.

The content of this narrative review was informed by the authors’ clinical and research expertise and supplemented by searches of PubMed and Embase databases. Literature was selected to provide a balanced overview of key concepts, emerging evidence, and areas of clinical relevance.

## 2 Pathophysiology

**FIGURE 1:**
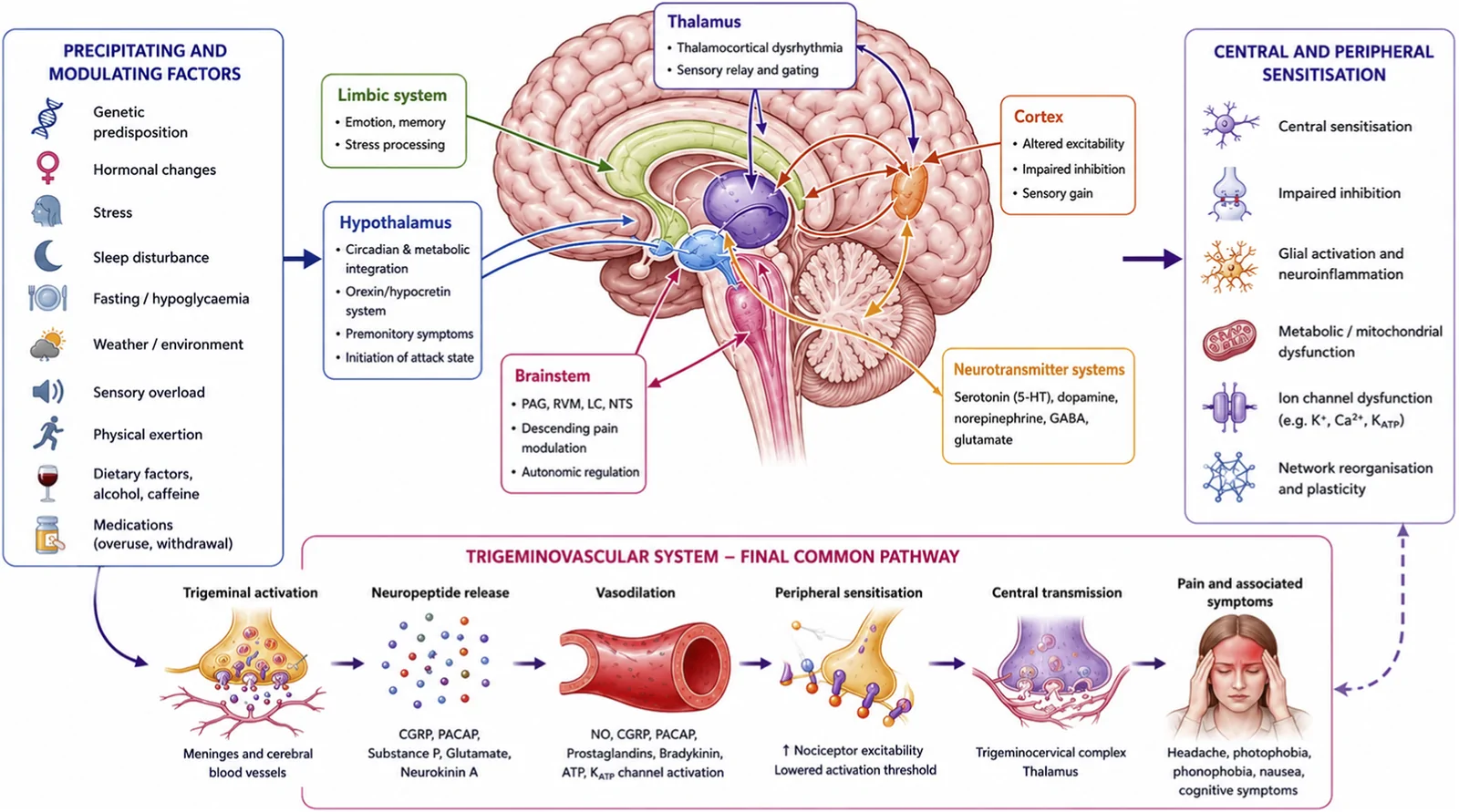
Diagram summarising key factors, neuroanatomical areas, and signalling molecules implicated in migraine pathophysiology. 5-HT, 5-Hydroxytryptamine (Serotonin); ATP, Adenosine Triphosphate; Ca^2+^, Calcium Ion; CGRP, Calcitonin Gene-Related Peptide; GABA, Gamma-Aminobutyric Acid; K^+^, Potassium Ion; KATP, ATP-Sensitive Potassium Channel; LC, Locus Coeruleus; NTS, Nucleus Tractus Solitarius (Nucleus of the Solitary Tract); PAG, Periaqueductal Gray; PACAP, Pituitary Adenylate Cyclase-Activating Polypeptide; RVM, Rostral Ventromedial Medulla; TCC, Trigeminocervical Complex; NO, Nitric Oxide.

Current understanding of migraine has shifted from viewing the disorder as a purely meningeal vascular phenomenon to recognising it as a complex neural network disorder with secondary vascular changes. This was driven by observations that headache severity correlates poorly with the degree of meningeal vasodilation and that effective migraine therapies act independently of major vascular effects (Goadsby et al., 2017). Instead, several signalling mediators are understood to link peripheral trigeminovascular activation to central pain processing, integrating vascular, inflammatory, and neuronal mechanisms into a single unified disease model (Figure 1).

### 2.1 Neuroanatomical pathways

Although the exact mechanism of migraine origin remains unknown, genetic predisposition, abnormal neurotransmitter signalling, metabolic susceptibility, and dysfunction of thalamic, hypothalamic, and brainstem networks are understood to lower neuronal activation thresholds thereby impairing sensory gating and pain modulation (Goadsby et al., 2017). Central to this model is the abnormal regulation of descending pain control, overseen by the hypothalamus and brainstem structures, such as the periaqueductal gray (PAG) and dorsal pons. Furthermore, the trigeminal nucleus caudalis functions as a key relay through which trigeminovascular activity is integrated with central pain-processing networks. Prior to headache initiation, as many as 77% of migraine patients may experience premonitory symptoms such as fatigue, yawning, appetite changes, nausea, and mood changes (Laurell et al., 2016)—functions regulated by hypothalamic networks.

Multiple neuroimaging studies suggest that altered trigeminocervical-hypothalamic networks crucially underpin migraine pathogenesis, positioning migraine as a disorder of brain state switching rather than a single focal generator. Positron emission tomography (PET) imaging has shown initiation of spontaneous migraine attacks to coincide with increased blood flow to the hypothalamus, midbrain, and pons (Denuelle et al., 2007). PET imaging has also shown that increased blood flow during migraine attacks within the cingulate gyrus, auditory cortex, and visual cortex diminish after relief of headache, photophobia, and phonophobia via sumatriptan injection, with only rostral brainstem blood flow remaining persistently elevated (Weiller et al., 1995). Moreover, functional magnetic resonance (fMRI) imaging has shown that migraine attacks can be predicted by interictal spinal trigeminal nuclei signal intensities (Stankewitz et al., 2011). MRI has shown significantly reduced volumes of thalamic nuclei in patients suffering from migraine (Magon et al., 2015), with fMRI also identifying altered thalamic connectivity during migraine attacks (Amin et al., 2018). Network reorganisation and neuroplastic changes within nociceptive circuitry may further contribute to abnormal sensory information processing and migraine susceptibility. Therefore, abnormal thalamic sensory gating may amplify the transmission of visual, auditory, and somatosensory information to cortical networks, thereby promoting sensory hypersensitivity and lowering the threshold for attack generation (Younis et al., 2019).

Beyond subcortical and brainstem network dysfunction, migraine may also involve intrinsic cortical hyperexcitability, First described in 1944 (Leão, 1944), cortical spreading depression (CSD)—a self-propagating wave of neuronal and glial hyperactivity followed by suppressed activity—remains the best-supported model for aura generation. fMRI has shown that, during aura, blood oxygenation level-dependent (BOLD) signal changes originate in the extrastriate visual cortex (V3A) and propagate in CSD-like patterns across the visual cortex, mirroring the progression of visual symptoms (Hadjikhani et al., 2001).

### 2.2 Signalling mediators

Upon migraine initiation and activation of the trigeminovascular system, several key neuropeptides are understood to underpin heightened nociception. To contextualise their complementary roles in migraine signalling, these mediators are discussed below within three overlapping functional levels: sensory transmission; signal amplification; and homeostatic modulation.

At the first level, nociceptive signals are transmitted via meningeal trigeminal afferent neurones whose cell bodies reside within the trigeminal ganglion before projecting centrally to the trigeminal nucleus caudalis, with calcitonin gene-related peptide (CGRP) being released from activated trigeminal sensory afferents and contributing to both peripheral and central sensitisation processes (Goadsby et al., 2017). CGRP is, therefore, positioned as a core molecular mediator of trigeminovascular signalling and a key therapeutic target in migraine pathophysiology Additionally, other members of the calcitonin peptide family, such as amylin, may also contribute to migraine pathophysiology through overlapping receptor signalling pathways with CGRP, although their specific role remains less established (Goadsby et al., 2017). Furthermore, dysfunction of serotonergic (5-Hydroxytryptamine; 5-HT) transmission has also been implicated in trigeminovascular activation, cortical excitability, and vascular tone regulation, with reduced cerebral serotonin levels reported between migraine attacks (Deen et al., 2017).

At the second level, tachykinins, such as substance P and neurokinin A (NKA), enhance signal gain within trigeminovascular pathways. Migraine models have historically proposed tachykinins as key mediators of meningeal neurogenic inflammation. Although substance P and NKA activate meningeal neurokinin-1 (NK1) and neurokinin-2 (NK2) receptors, respectively, to induce plasma protein extravasation, vascular permeability, and mast cell activation in preclinical studies, these effects have not translated into convincing evidence for a pathogenic role in human migraine (Goadsby et al., 2017). Although CGRP has also been incorporated into historical models of neurogenic inflammation, its actions appear distinct from classical inflammatory mediators such as substance P. Human mast cells have since been shown not to express CGRP receptors (Eftekhari et al., 2013). NK1 receptor expression on human mast cells (Kulka et al., 2008) highlights a potential neuroimmune interface within the meninges to modulate nociceptive signalling, although this pathway’s contribution to spontaneous migraine remains unclear. Human mast cells additionally express vasoactive intestinal peptide receptor 2 (VPAC2) receptors which are activated by pituitary adenylate cyclase-activating polypeptide (PACAP) and vasoactive intestinal polypeptide (VIP) (Kulka et al., 2008). PACAP and VIP infusions have been shown to induce migraine attacks in migraineurs (Ghanizada et al., 2020; Pellesi et al., 2021), thereby supporting a neuroimmune contribution to migraine pathophysiology, although this mechanism appears distinct from the classical substance P-mediated neurogenic inflammation model. Alongside neuroimmune mechanisms, vasoactive mediators represent another layer of signal amplification within the trigeminovascular system, contributing to trigeminal activation, meningeal nociception, and central pain processing. Although vascular dilation is no longer considered the primary origin of migraine pain, CGRP and other vasodilatory mediators, such as nitric oxide (NO) and ATP-sensitive potassium (K_ATP_) channel and glutamatergic signalling, contribute to the amplification and modulation of trigeminovascular activity. This is evidenced by NO prodrugs, such as glyceryl trinitrate, having been shown to induce migraine attacks in susceptible individuals (Christiansen et al., 2000), an effect which can be primed by dural protease-activated receptor-2 (PAR2) agonists (Mason et al., 2023).

Finally, at the third level, PACAP represents a bridge between peripheral signal modulation and central homeostatic regulation. As a widely distributed neuromodulatory peptide, PACAP is expressed within the trigeminal ganglion, parasympathetic ganglia, brainstem, hypothalamus, and cortical networks. This extensive anatomical distribution enables PACAP to influence multiple processes relevant to migraine susceptibility, including sleep, stress responses, feeding behaviours, and circadian regulation, while modulating downstream trigeminovascular activation (Holland et al., 2018). Produced by the hypothalamus, Orexin (also known as hypocretin), has also attracted significant attention due to its ability to influence arousal, sleep-wake regulation, energy balance, stress responses, autonomic function, and pain processing (Strother et al., 2018; Stanyer et al., 2024). Orexin has also been shown to attenuate neurogenic dural vasodilation and inhibit presynaptic CGRP release from trigeminal neurones (Holland et al., 2005). Therefore, orexin may contribute to migraine susceptibility by regulating hypothalamic control of trigeminovascular excitability and nociceptive processing.

## 3 CGRP-targeted therapies

CGRP is a 37-amino-acid neuropeptide with two isoforms: α-CGRP, the predominant form implicated in migraine, and β-CGRP which is more commonly expressed within the enteric nervous system. Its molecular structure consists of an N-terminal disulfide-bonded ring between residues 2 and 7 and a C-terminal amidated region, both essential for receptor binding and biological activity. CGRP exerts its effects via a class B G protein–coupled receptor (GPCR) complex consisting of three subunits: calcitonin receptor-like receptor (CLR); receptor activity-modifying protein 1 (RAMP1); and an intracellular signalling accessory protein called receptor component protein (RCP) (Goadsby et al., 2017). This receptor complex predominantly signals via Gs-mediated activation of adenylyl cyclase and subsequent increased intracellular concentrations of the secondary messenger cyclic adenosine monophosphate (cAMP). Within the trigeminovascular system, CGRP is predominantly stored in unmyelinated C-fibre sensory neurones, whereas its receptor components are expressed on adjacent Aδ fibres and vascular smooth muscle cells (Eftekhari et al., 2013). Due to an established association between elevated CGRP levels and migraine attacks, modulation of CGRP signalling has emerged as a key therapeutic target for the treatment and prophylaxis of migraine.

### 3.1 CGRP receptor antagonists (“gepants”)

The first CGRP receptor antagonist, BIBN4096BS, now known as olcegepant, was first described in 2000 when intravenous infusions given to marmoset monkeys were shown to cause a dose-dependent inhibition of facial vasodilatory responses evoked by trigeminal ganglion electrical stimulation (Doods et al., 2000). Since this finding, research into the class of drug, colloquially known as a “gepants,” boomed and the drugs are now available in oral and intranasal regimens. Though the drugs work primarily via antagonism of the CGRP receptor complex (CLR/RAMP1/RCP), the drugs also have high affinity for other members of the “calcitonin family,” most notably Amylin 1 (AMY1) receptors but also, with much lower binding affinity, to calcitonin receptors, amylin 3 (AMY3) receptors, and adrenomedullin 1 and 2 (AM1, AM2) receptors (Pan et al., 2020; Moore et al., 2024).

Currently, four gepants have been approved by the United States’ Food and Drug Administration (FDA): rimegepant (orodispersible tablet), atogepant (oral tablet), ubrogepant (oral tablet), and zavegepant (intranasal spray). Rimegepant has also been approved by The National Institute for Health and Care Excellence (NICE) for the treatment of migraine unresponsive to two triptan treatments (TA919) (National Institute for Health and Care Excellence, 2023b) and as a prophylactic mediation in episodic migraine where at least three other preventative medications have not worked (TA906) (National Institute for Health and Care Excellence, 2023a). Atogepant is currently only approved by NICE for the latter scenario (TA973) (National Institute for Health and Care Excellence, 2024); however, further guidance is in development for the drug to also be used as an acute migraine treatment (GID-TA11817) (National Institute for Health and Care Excellence, 2026).

Regarding the acute treatment of migraine, a systematic review found that across 7,198 patients, a single 75 mg oral dose of rimegepant given to patients during migraine attacks had a higher likelihood of evoking pain relief (relative risk (RR) 1.35; 95% confidence interval (CI): 1.28–1.42) and pain freedom (RR 1.77; 95% CI: 1.5–2.1) at 2-h compared to placebo (Khodeer et al., 2026). However, another systematic review which evaluated data for 89,445 patients found that rimegepant and ubrogepant both had inferior efficacy for pain relief and pain freedom at 2 hours compared to triptan therapies (Karlsson et al., 2024). This suggests that gepant drugs should not replace initial migraine treatment lines but rather may have a role in the treatment of patients who are unresponsive to initial treatment approaches. A cost-benefit analysis, however, found that neither rimegepant, ubrogepant, nor zavegepant were cost-effective for the acute treatment of migraine when looking at quality adjusted life year improvements compared to treatment with paracetamol, NSAIDs, and triptans (Gokhale and Villa Zapata, 2025). Though it should be noted that cost-effectiveness findings are healthcare system-dependent and may not translate globally.

Evidence for gepants in migraine prophylaxis is currently limited and diverse. Rimegepant offers promised as a preventative medicine in episodic migraine. Alternate-day rimegepant 75 mg administration has demonstrated preventive efficacy in patients with episodic migraine, including individuals with higher-frequency migraine patterns, with significant reduction in mean monthly migraine days in the third month of treatment (Croop et al., 2021). However, evidence of prophylactic benefit of gepant therapy in CM is much more limited. A systematic review of data for 14,725 patients with CM found that atogepant reduced monthly headache days by −2.10 days (CI: −3.06 to −1.14) while rimegepant showed, with moderate certainty, no statistically significant benefit (−1.20 days; CI: −2.59 to 0.19) (Khalili et al., 2026a). Another systematic review of 4,325 patients, with a mixture of EM and CM diagnoses, found a small benefit over placebo in reducing mean monthly migraine days (difference −0.39 days; 95% CI: −0.45 to −0.33) (Shaukat et al., 2025). However, gepants have been shown to have benefit in reducing medication overuse; one retrospective observational study found that 82% of patients with either chronic or high-frequency episodic migraine who received oral atogepant reported a reduction in their triptan use, with almost half of patients reporting a reduction of 60% or more (Guerzoni et al., 2026). As ubrogepant and zavegepant have half-lives between 5 and 7 h (Scott, 2020; Dhillon, 2023), the drugs have limited applicability in migraine prophylaxis.

Gepants are generally well-tolerated; however, adverse effects include nausea, vomiting, and constipation (Karlsson et al., 2024; FAERS database, 2026). Atogepant has overall adverse event rates comparable to placebo and few treatment discontinuations due to adverse effects (Ailani et al., 2021). The most common adverse events associated with atogepant were constipation (6.9%–7.7%) and nausea (4.4%–6.1%), while serious adverse events are rare and no evidence of the hepatotoxicity reported with earlier generations of gepants has been observed (Ailani et al., 2021). These gastrointestinal effects observed clinically are supported by preclinical studies, where olcegepant was shown to attenuate CGRP-induced diarrhoea in mice, suggesting a potential role for CGRP signalling in intestinal motility (Kaiser et al., 2017).

### 3.2 Monoclonal antibodies

Both Anti-CGRP (fremanezumab, galcanezumab, and eptinezumab) and Anti-CGRPR (erenumab) antibodies have been developed. All four antibody therapies are recommended by NICE for the prevention of migraine in patients with at least four migraine days per month, where there has been a poor response to at least three other preventive medicines, and if the therapy is provided under the Simple Discount Patient Access Scheme’s commercial agreements (National Institute for Health and Care Excellence, 2022; National Institute for Health and Care Excellence, 2023c; National Institute for Health and Care Excellence, 2020; National Institute for Health and Care Excellence, 2021).

All antibody therapies available in subcutaneous preparations except eptinezumab which is given intravenously. Injection regimens are commonly either 100 mg monthly or, 300 mg given every 3 months. Each treatment should be stopped if after 12 weeks migraine frequency does not reduce by at least 50% in patients with EM or by at least 30% in patients with CM (National Institute for Health and Care Excellence, 2022; National Institute for Health and Care Excellence, 2023c; National Institute for Health and Care Excellence, 2020; National Institute for Health and Care Excellence, 2021). Cell models have shown these two antibody classes block both CGRP-induced cAMP signalling and, at much higher concentrations, CGRP receptor internalisation; neither of these effects were seen in the absence of CGRP (Manoukian et al., 2019).

A systematic review of data for 9,123 patients found that 240 mg doses of galcanezumab had the highest efficacy in reducing monthly migraine days, 200 mg doses of fremanezumab had the highest likelihood of a ≥50% reduction in migraine days, and 28 mg doses of erenumab had the best safety profile (Shakir et al., 2026). Overall, fremanezumab offered the best balance of both efficacy and safety (Shakir et al., 2026). Another systematic review of 7,281 patients who failed to respond to prior preventive migraine treatments found that eptinezumab was superior to both atogepant and also other monoclonal antibodies in reducing monthly migraine days (Khalili et al., 2026b). Eptinezumab similarly outperformed both gepants and BoNT-A injections in a systematic review of 14,725 patients with CM (Khalili et al., 2026a).

However, there is significant heterogeneity in the methodology of studies investigating CGRP monoclonal antibodies for migraine. A systematic review of forty-six studies found that eight (17.4%) did not define a primary endpoint, nineteen (41.3%) formulated exclusion criteria, and only 31 (67%) used a fixed starting dose for each participant (Vandenbussche et al., 2023). Monoclonal antibodies are also very expensive to administer; the highest price for one dose is £4,050 for a single 300 mg eptinezumab 3-monthly injection, though the NHS has negotiated a confidential commercial discount (National Institute for Health and Care Excellence, 2023c).

Adverse effects are similar to that for gepants (i.e., nausea, vomiting, and constipation) and, additionally, hypertension, ischaemic stroke, and alopecia (Russo et al., 2026). Similar to gepants, monoclonal antibodies have been shown to block CGRP-induced diarrhoea, and have also been suggested to have a potential role in managing CGRP-mediated *clostridium difficile* colitis (Kaiser et al., 2017).

### 3.3 Combined therapies

There is increasing evidence that combining CGRP-targeted therapies offers a more efficacious approach than monotherapy with either agent. This has been shown to be a safe and well-tolerated approach (Alsaadi et al., 2024). Rimegepant 75 mg dosing may be effective strategy for the acute management of breakthrough migraine attacks in patients taking erenumab, fremanezumab, or galcanezumab prophylactic therapy (Mullin et al., 2020; Berman et al., 2020). Ubrogepant 50 and 100 mg doses have similarly been shown to help achieve meaningful pain relief in patients receiving monoclonal antibody therapy (Lipton et al., 2024).

Combining gepants with monoclonal antibodies as a dual-preventative strategy has also been shown to cause a significant decrease in monthly headache days without any increase in adverse events (Graves et al., 2026). Moreover, dual-gepant therapy with ubrogepant and atogepant co-administration has been shown to be safe and well-tolerated (Blumenfeld et al., 2023); however, there is presently limited evidence on the efficacy of this approach.

CGRP monoclonal antibodies are also an efficacious add-on prophylactic therapy for patients with CM already receiving BoNT-A injections, with 111 of 153 (72.5%) patients reporting a decrease in either monthly headache days or headache pain severity (Cohen et al., 2021). Ubrogepant 50 and 100 mg doses have also been shown to achieve meaningful pain relief at 2-h in 53.3% of patients receiving BoNT-A migraine prophylaxis (Manack Adams et al., 2023). A similar benefit was also seen in patients receiving BoNT-A and CGRP monoclonal antibody combined prophylaxis (Lipton et al., 2024). It should also be noted that prior success or failure to CGRP monoclonal antibody and BoNT-A injections is predictive of gepant treatment response and patient satisfaction (Chiang et al., 2021).

**FIGURE 2:**
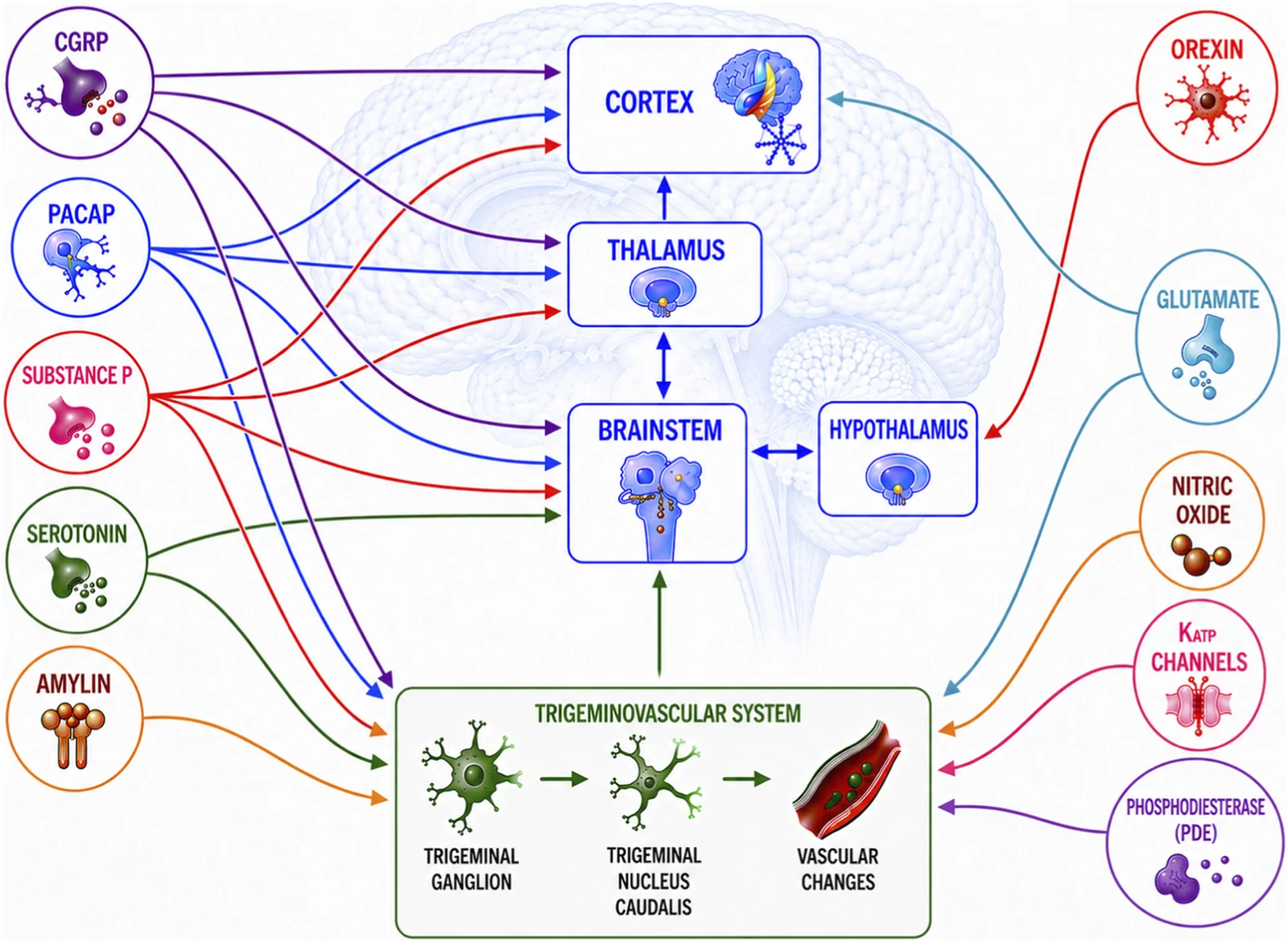
Diagram summarising emerging pharmacological targets for the treatment of migraine and their principal sites of action. K_ATP_, ATP-sensitive potassium channel; PDE, Phosphodiesterase.

Overall, CGRP-targeted therapies offer a novel and bespoke migraine treatment with promising benefits for both acute migraine management and prophylactic therapy (Figure 2). The American Headache Society recently published a position statement recommending that CGRP-targeted therapies should be used as first-line agents in migraine prophylaxis (Charles et al., 2024). Regarding acute migraine management, current efficacy and limited cost-benefit data suggests that CGRP-targeted therapies are better placed as third and fourth line treatments for patients non-responsive to initial treatment lines.

## 4 PACAP-targeted therapies

PACAP is a pleiotropic neuropeptide belonging to the secretin/glucagon family with two predominant biologically active isoforms: PACAP-38 and PACAP-27. Similar to CGRP, PACAP exerts its effects via three class B GPCR’s: the pituitary adenylate cyclase-activating polypeptide type I receptor (PAC1) which shows high selectivity and affinity for PACAP; and the vasoactive intestinal peptide receptors 1 and 2 (VPAC1 and VPAC2) which have affinity for both PACAP and VIP. These three receptors act primarily via Gs-mediated signalling; however, PAC1 can also couple to Gq-mediated phospholipase C signalling in a tissue-dependent manner (Holland et al., 2018).

Migraine-like attacks have been shown to be induced by both CGRP (63%) and PACAP (72%) infusions, with the latter much more likely to cause premonitory symptoms (48% vs. 9%) (Guo et al., 2016). This effect had also been seen in patients receiving PACAP infusions while also on Eptinezumab prophylactic therapy (Al-Karagholi et al., 2025), suggesting PACAP plays a crucial role in migraine pathophysiology independent to that of CGRP. This evidence, along with the aforementioned success of CGRP-targeted therapies, has stimulated growing interest in PACAP as a therapeutic target for migraine therapy.

Animal models have shown neuronal PAC1 receptors to mediate delayed activation and sensitisation of trigeminocervical neurones. VIP and PACAP-38 infusions induce transient meningeal vasodilation via VPAC2 receptors, without concurrent activation of central trigeminovascular neurones; however, PACAP-38 infusions additionally lead to delayed activation and sensitisation of these neurones, consistent with its delayed induction of migraine-like headache (Akerman and Goadsby, 2015). Intravenous administration of a PAC1 receptor antagonist reduced meningeal vasodilation associated with dural trigeminovascular nociception, whereas intracerebroventricular administration inhibited nociception-evoked activity within central trigeminovascular neurones, supporting the conclusion that migraine-related mechanisms are primarily centrally mediated, likely within the trigeminocervical complex, rather than driven by the dura mater or its primary afferent innervation (Akerman and Goadsby, 2015). In another rodent model, PAC1 receptor blockade with a monoclonal antibody has been shown to inhibit stimulus-evoked trigeminocervical complex activity (Hoffmann et al., 2020). Moreover, PAC1 antagonism has been shown to reverse chronic allodynia and inhibit opiate-exacerbated CSD, suggesting a potential role for PAC1-targeted therapeutics in medication overuse headaches (Bertels et al., 2023).

There is currently mixed evidence that these benefits translate to human populations. A phase 2 randomised controlled trial (RCT), which enrolled adult migraineurs who had failed to respond to previous preventative therapies, found a PACAP ligand-directed monoclonal antibody (Lu AG09222) to significantly outperform placebo in reducing migraine frequency over the 4-week period following administration (Ashina et al., 2024). However, another ligand-directed monoclonal antibody (LY3451838) offered no meaningful benefit over placebo for migraine prevention in a patients with treatment-resistant EM and CM (Johnson et al., 2025). Similarly, another phase 2 RCT found that the receptor-directed PAC1 monoclonal antibody (AMG 301) to have no statistically significant benefit in preventing migraine within a mixed EM and CM patient cohort (Ashina et al., 2021).

Cellular studies have shown VPAC1 and VPAC2 receptor antibodies to have successfully high specificity and affinity for receptors (Tasma et al., 2022); however, currently, there are no registered clinical studies investigating antibody nor antagonist therapies directed against VPAC1/2 receptors nor VIP signalling. Further research into both the role of PACAP in migraine pathophysiology and the efficacy of PACAP-targeted therapeutics within human migraine populations is needed.

## 5 Amylin-targeted therapies

Amylin (islet amyloid polypeptide) is a 37-amino-acid peptide hormone. A member of the calcitonin peptide family, it is co-secreted pancreatic β-cells with insulin to regulate satiety, glucose homeostasis, and gastric emptying. Interictal blood concentrations of amylin have been shown to be raised in migraine patients, with higher levels in those with CM rather than EM (Irimia et al., 2021). Moreover, amylin is structurally similar to CGRP with an N-terminal disulfide bond between residues 2 and 7 and a C-terminal amidated region. Due to this similarity and the identification that CGRP activates AMY1 and AMY3 receptors, amylin signalling has received increasing attention as a novel therapeutic target in migraine.

A double-blind randomised study showed that, in a cohort of patients with migraine without aura, infusions of pramlintide, an amylin analogue, stimulated headache in 88% and migraine-like attacks in 41% (Ghanizada et al., 2021). These values for CGRP were 97% and 56% respectively (Ghanizada et al., 2021). Additionally, a rodent study has shown that both amylin and CGRP cause evoke dose-dependent squint responses indicative of pain, with female mice exhibiting more sensitive responses than males (Rea et al., 2022). Additionally, amylin knockout mice exhibit reduced nociceptive responses (Gebre-Medhin et al., 1998).

An amylin receptor antagonist, AC187, has been developed and shown to accelerate gastric emptying and increase glucagon secretion in rats (Gedulin et al., 2006). However, there is presently no clinical evidence for amylin-targeted therapies in migraine nor any registered clinical trials.

## 6 Neurokinin-targeted therapies

Neurokinins, also known as tachykinins, are a family of neuropeptides that includes substance P, neurokinin A, and neurokinin B. They act through three G-protein coupled receptors: NK1, NK2, and neurokinin-3 (NK3) receptors, with substance P preferentially activating NK1, neurokinin A preferentially activating NK2, and neurokinin B preferentially activating NK3 (Alpay et al., 2025). Substance P antagonists generally refer to agents that inhibit substance P signalling, most commonly through NK1 antagonism, although substance P may also exert biological effects through non-NK1 mechanisms (Alpay et al., 2025). Clinically, NK1 antagonists have established roles in other settings, particularly as antiemetics, but this has not translated into approved migraine therapy (Sharun et al., 2021).

Trigeminal afferents innervating the dura and cranial vasculature contain several vasoactive neuropeptides, including substance P, neurokinin A, and CGRP (Alpay et al., 2025; Beattie et al., 1995). Following trigeminovascular activation, substance P is released from primary afferent neurones and activates NK1 receptors expressed on peripheral and central pain-processing structures. Preclinical studies demonstrated that substance P signalling can promote meningeal vasodilation, plasma protein extravasation, mast cell degranulation, immune cell recruitment, and enhanced trigeminal nociceptive signalling within animal studies providing a strong experimental rationale for the development of NK1 antagonists as non-vasoconstrictive migraine therapies. Moreover, early pharmacological studies showed that selective NK1 antagonists inhibit substance P-mediated cranial vasodilation and plasma protein extravasation in experimental models, supporting their potential role in migraine therapy (Beattie et al., 1995; May and Goadsby, 2001). Although it is now understood that neurogenic inflammation mechanisms observed in animal models do not represent primary drivers of human migraine pathophysiology, these experimental findings established an important framework for investigating substance P-mediated pathways as a potential pharmacological target in migraine.

However, clinical translation of these findings has been disappointing. In an acute migraine crossover study, oral lanepitant (an NK1 antagonist) at 30, 80, and 240 mg failed to improve 2-h headache relief compared with placebo, although adverse events were similar across treatment arms (Goldstein et al., 1997). In a separate 12-week preventive trial, lanepitant 200 mg daily did not meet its predefined primary endpoint for a 50% or greater reduction in monthly migraine days across months 1–3, although a secondary month-3 signal was observed, with 40.6% of lanepitant-treated patients achieving response compared with 17.4% on placebo (Goldstein et al., 2001a). Other NK1 antagonists were similarly disappointing: GR205171, given intravenously at 25 mg, failed to improve 2-h headache relief in acute migraine; L-758,298 showed no benefit over placebo, with 2-h headache relief rates of 33% versus 52% for placebo at the 60 mg dose; and RPR100893, also known as dapitant, tested at oral doses of 1, 5, and 20 mg in 139 patients, produced no significant improvement in headache intensity or 2-h headache response compared with placebo (Alpay et al., 2025; Pellesi and Edvinsson, 2025). These findings suggest that NK1 antagonism alone is insufficient as an acute or preventive migraine strategy, despite generally acceptable tolerability in early trials (Alpay et al., 2025; Goldstein et al., 1997; Goldstein et al., 2001a; Pellesi and Edvinsson, 2025).

The discrepancy between preclinical evidence and negative clinical trials has several possible explanations. NK1 antagonism alone may not fully inhibit substance P signalling, particularly if migraine-relevant effects occur through central pathways, receptor internalisation, endosomal signalling, neuroimmune mechanisms, or non-NK1 targets (Alpay et al., 2025; Pellesi and Edvinsson, 2025). Substance P may also interact with other migraine-relevant neuropeptide systems, including CGRP and PACAP, or act through additional pathways beyond NK1, meaning that single-receptor blockade may be insufficient (Alpay et al., 2025; Pellesi and Edvinsson, 2025). This has led to renewed interest in ligand-directed approaches, including the theoretical possibility of substance P antibodies or broader strategies targeting downstream neuroimmune signalling. However, unlike CGRP monoclonal antibodies, there are currently no approved substance P antibodies or neurokinin receptor antibody therapies for migraine, and no human migraine trial has demonstrated clinical efficacy for this approach (Pellesi and Edvinsson, 2025).

Recent human provocation studies have reopened interest in substance P as a migraine-relevant mediator. Intravenous substance P has been shown to induce headache and superficial temporal artery dilation in healthy adults, supporting a role for substance P in head pain and vascular modulation (Al-Khazali et al., 2025). A subsequent preprint study reported that intravenous substance P triggered migraine attacks without aura in adults with migraine, although these data should be interpreted cautiously until full peer-reviewed publication (Al-Khazali et al., 2026a).

Overall, neurokinin receptor antagonists represent a biologically plausible but clinically unsuccessful class of migraine therapeutics to date. Current evidence does not support NK1 antagonists as effective acute or preventive migraine treatments, and antibody-based approaches against substance P remain theoretical rather than clinically validated. Nevertheless, recent substance P provocation data suggest that the pathway may warrant re-evaluation, particularly through ligand-level targeting, combined neuropeptide blockade, or approaches addressing non-NK1R and neuroimmune mechanisms.

## 7 Serotonin-targeted therapies

Serotonergic signalling has long been implicated in migraine pathophysiology, with human neuroimaging and electrophysiological studies supporting altered central serotonin neurotransmission across the migraine cycle (Deen et al., 2017). PET studies have provided evidence of abnormal serotonin activity in migraine, with evidence of increased brain serotonin synthesis and altered serotonin receptor binding between attacks (Chugani et al., 1999; Deen et al., 2018). Serotonin also exerts vascular and trigeminovascular effects through receptor-specific mechanisms. Activation of vascular 5-HT_2B_ and 5-HT_7_ receptors has been proposed to promote cranial vasodilation and trigeminovascular afferent activation, whereas 5-HT_1B_ receptor activation can constrict meningeal vessels and 5-HT_1D/1F_ receptor activation may inhibit neurogenic inflammation and nociceptive transmission (Hamel, 1999). These serotonergic mechanisms intersect with calcitonin gene-related peptide (CGRP) biology, as trigeminovascular activation leads to CGRP release, neurogenic vasodilation, and pain transmission, while serotonergic migraine agents can modulate CGRP-related signalling (Aggarw et al., 2012).

This biological rationale historically informed the use of serotonin antagonists for migraine prevention, including agents such as methysergide and pizotifen. Early comparative studies suggested that serotonin antagonism could reduce migraine frequency, supporting the concept that serotonergic dysregulation contributes to attack susceptibility (Lance et al., 1970). However, their role in contemporary practice has diminished, partly due to tolerability concerns and adverse effects, moreover a shift toward more selective, receptor-specific and pathway-directed approaches.

Ditans represent a modern serotonergic strategy that is pharmacologically distinct from both historical serotonin antagonists and triptans. They are selective serotonin 5-HT_1F_ receptor agonists developed for the acute treatment of migraine (Mitsikostas et al., 2023). The principal agent in this class is lasmiditan, a centrally penetrant 5-HT_1F_ receptor agonist used for acute migraine with or without aura (Berger et al., 2020). Unlike triptans, which act mainly through 5-HT_1B/1D_ receptors and may produce cranial vasoconstriction, lasmiditan does not rely on vascular smooth muscle constriction (Hamel, 1999; Mitsikostas et al., 2023; Berger et al., 2020). This provides a non-vasoconstrictive acute treatment option for patients in whom triptans are contraindicated, ineffective, or poorly tolerated because of cardiovascular risk.

Mechanistically, 5-HT_1F_ receptor activation is thought to reduce trigeminovascular nociceptive signalling by inhibiting trigeminal pathway activation and modulating neuropeptide-mediated transmission (Mitsikostas et al., 2023; Berger et al., 2020). This places ditans at the interface between older serotonergic models of migraine and contemporary neuropeptide-centred frameworks involving CGRP and trigeminovascular activation. Clinical studies support lasmiditan’s efficacy for acute migraine endpoints, including 2-h pain freedom and relief of the most bothersome associated symptom (Berger et al., 2020). However, comparative evidence suggests that although lasmiditan is superior to placebo, several triptans, including eletriptan, rizatriptan, sumatriptan, and zolmitriptan, have stronger overall efficacy profiles for acute migraine treatment (Karlsson et al., 2024). Contemporary reviews therefore position ditans as useful alternatives rather than replacements for triptans, particularly in patients with vascular contraindications or poor triptan tolerability (Burch and Rittenberg, 2025; Mínguez-Olaondo et al., 2024).

The main limitation of lasmiditan is its central nervous system adverse-effect profile. Dizziness, somnolence, fatigue, paraesthesia, and impaired alertness are commonly reported, reflecting its central penetrance (Berger et al., 2020). Safety reviews also highlight the need to consider sedation, driving impairment, concomitant centrally acting drugs, and the limited evidence base for drug interactions when ditans are used alongside other migraine therapies (Al-Hassany and MaassenVanDenBrink, 2021). Other 5-HT_1F_ agonists have not progressed to clinical approval, although LY334370 showed dose-dependent efficacy in an early randomised trial and lacked clinically relevant vasoconstrictive effects (Goldstein et al., 2001b). Its development was discontinued because of compound-specific safety concerns during longer-term animal exposure. Another ditan, LY344864, has shown potential in preclinical models by reducing trigeminovascular nociceptive signalling without causing vasoconstriction, although it has remained an experimental research compound and has not progressed to approved clinical use (Goldstein et al., 2001b; Rubio-Beltrán et al., 2018).

Lasmiditan 50, 100, and 200 mg oral doses have also shown promise in treating perimenstrual migraine attacks, with one in three patients who received 200 mg doses reporting being pain-free at 2 hours (MacGregor et al., 2022). Additionally, a Bayesian network meta-analysis of 4,304 patients with menstrual migraine found that lasmiditan outperformed both rizatriptan and placebo in achieving sustained pain freedom at 24 h; however, there was no observable difference between ditan and triptan therapies in achieving pain freedom nor pain relief at 2 hours (Song et al., 2025).

Overall, serotonergic modulation remains therapeutically relevant in migraine, but the field has shifted from broad serotonin antagonism toward receptor-specific approaches such as 5-HT_1F_ agonism.

## 8 Orexin-targeted therapies

Orexin-A and orexin-B are derived from the precursor prepro-orexin and act through two G-protein coupled receptors, OX1R and OX2R, which differ in ligand affinity and anatomical distribution (Strother et al., 2018; Stanyer et al., 2024). Whilst activation of either receptor is excitatory, the OX1R is selective for OXA, whereas OX2R is nonselective for OXA and OXB. However, OXB has a 10-fold higher affinity for the OX2R than the OX1R (Lang et al., 2004). Orexin-producing neurones are concentrated in the lateral, perifornical, and dorsomedial hypothalamus, but project widely to migraine-relevant regions including the trigeminal nucleus caudalis, locus coeruleus, dorsal raphe nucleus, periaqueductal grey, thalamus, and cortex (Lang et al., 2004; Stanyer et al., 2024).

Neuroimaging studies have demonstrated hypothalamic activation during both the premonitory and headache phases of migraine, including in spontaneous attacks and experimentally induced migraine (Denuelle et al., 2007; Schulte and May, 2016; Maniyar et al., 2014). In addition, altered functional connectivity between the hypothalamus and regions involved in pain processing has been detected before the clinical onset of migraine, suggesting that hypothalamic network dysfunction may precede attack generation (Stankewitz et al., 2021; Moulton et al., 2014; Lerebours et al., 2019; Coppola et al., 2020; Messina et al., 2022; Meylakh et al., 2020).

Clinical observations linking migraine prodrome symptoms, such as fatigue, yawning, appetite change, mood disturbance, and sleep disruption, to hypothalamic activation support a role for orexinergic dysfunction early in attack generation (May et al., 2019; Gollion et al., 2022). Preclinical studies further suggest that orexin signalling can attenuate neurogenic dural vasodilation, modulate trigeminal nociception, and increase the threshold for CSD, implicating it in both migraine with and without aura (Holland et al., 2005; Hof et al., 2015). Rather than acting as a direct pain mediator, orexin may function as an upstream regulator of migraine susceptibility by influencing arousal state, autonomic balance, sensory gain, and nociceptive threshold within broader hypothalamic–trigeminovascular networks (Hof et al., 2015; Salinas-Abarca et al., 2025).

Therapeutically, the orexin system has emerged as a potential pharmacological target. However, clinical translation into migraine therapy has thus far been disappointing. Higher cerebrospinal fluid (CSF) concentrations of orexin have been reported in a case-control study looking at chronic migraines in human studies (Sarchielli et al., 2008). However, this finding is not consistent within the literature (Özaydin et al., 2016).

Dual Orexin Receptor Antagonists (DORAs) have equal affinity for both orexin receptors. DORA-12, antagonises an orexin-induced calcium increase in cells expressing OXR1 or OXR2 (Winrow et al., 2012). One *in vivo* study found that DORA-12 attenuated the induction of migrainous activity in rats caused by middle meningeal artery and parietal cortical stimulation (Hof et al., 2015). From a therapeutic standpoint, another orexin receptor antagonist, filorexant (MK-6069), has good oral bioavailability, readily crosses the blood brain barrier, and binds orexin receptors with high affinity (Winrow et al., 2012). A randomised controlled trial evaluating the dual orexin receptor antagonist filorexant (MK-6069) did not show significant benefit for migraine prevention, despite promising preclinical data (Chabi et al., 2015). This lack of efficacy may, however, relate to pharmacokinetic and dosing limitations, including nocturnal administration and the relatively short half-life of filorexant of approximately 3–5 h (Chabi et al., 2015; Goadsby, 2015). Suvorexant (MK-4305), another DORA medication with a half-life of 12 h, has been approved for treatment of insomnia and has also been shown to prevent, but not treat, migraine attacks in male mice (Kopruszinski et al., 2024).

The clinical efficacy of DORAs for the treatment of migraine remains unclear. A rat model showed that orexin-A microinjection into the posterior hypothalamus suppressed dural-evoked activation of the trigeminal system, while orexin-B enhanced this response (Bartsch et al., 2004). Systemic administration of orexin-A also demonstrated antinociceptive effects in a model of neurogenic dural vasodilation, orexin-A, but not orexin-B, inhibited vasodilatory responses (Holland et al., 2005; Holland et al., 2006). These effects appeared to be mediated through OX1R activation, as they were abolished by selective OX1R antagonism.

Nonetheless, the association between hypothalamic dysfunction, sleep disturbance, and migraine attack generation continues to support investigation of orexin-related pathways (Stanyer et al., 2024; Lang et al., 2004). Future therapeutic strategies may involve selective receptor modulation, network-based interventions, or a combination of approaches targeting multiple hypothalamic neuropeptide systems simultaneously.

Collectively, current evidence suggests that orexin occupies a strategic position at the interface between hypothalamic homeostatic regulation and trigeminovascular activation. Although its precise role in migraine initiation remains incompletely defined, orexin represents a biologically plausible target within emerging network-based models of migraine and may contribute to future generations of mechanism-based therapeutics (Stanyer et al., 2024; Lang et al., 2004). However, preclinical findings are discordant: orexin-A has been shown to produce antinociceptive effects via OX1R activation (Holland et al., 2006), while DORAs may attenuate migrainous activity (Hof et al., 2015). These seemingly contradictory findings highlight the need for further mechanistic studies to clarify the role of orexinergic signalling in migraine and to guide possible therapeutic development.

## 9 Potassium channel-targeted therapies

Several potassium channel families are understood to play a role in migraine, including ATP-sensitive potassium (K_ATP_), “big” potassium (BKCa), voltage-gated potassium (K_v_), and inwardly rectifying potassium (K_ir_) channels (Al-Karagholi, 2023). Among these, K_ATP_ channels have emerged as the most extensively target for therapeutic development owing to their roles as downstream effectors of migraine signalling pathways, including those activated by neuropeptides such as CGRP (Al-Karagholi et al., 2017). K_ATP_ channels consist of four inwardly rectifying potassium channel (K_ir_6.x) pore-forming subunits and four regulatory sulfonylurea receptor (SUR) subunits (Al-Karagholi et al., 2017). Four principal channel subtypes have been identified: K_ir_6.1/SUR2B, K_ir_6.2/SUR1, K_ir_6.2/SUR2A and K_ir_6.2/SUR2B, each with distinct tissue distributions and physiological functions (Al-Karagholi et al., 2017; Clement et al., 2022). The vascular K_ir_6.1/SUR2B KATP channel subtype has received particular attention due to its expression in smooth muscle tissue and its ability to couple intracellular signalling pathways to changes in vascular tone (Al-Karagholi, 2023).

Intravenous 0.05 mg/min infusions of levcromakalim, a K_ir_6.1/SUR2B-selective K_ATP_ channel opener, were shown in a double-blind, placebo-controlled crossover study to trigger migraine attacks in 14 (82%) patients with only (6%) patient reporting a migraine attack after receiving a saline placebo infusion (Al-Karagholi et al., 2021a). Ten (59%) patients developed migraine with aura after receiving levcromakalim compared to zero patients in the placebo arm (Al-Karagholi et al., 2021a). In contrast, NN414, a selective K_ir_6.2/SUR1 channel opener induced migraine attacks in only two (16.6%) patients compared to one (8.3%) patient in the placebo arm (Kokoti et al., 2024). This further reinforces the K_ir_6.1/SUR2B K_ATP_ channel as a specific therapeutic target candidate for future migraine therapies.

However, translation of these observations into effective pharmacological therapies has remained challenging. Oral administration of 10 mg glibenclamide, a sulfonylurea drug which inhibits K_ATP_ channels, was shown in a three-way crossover study to delay the onset, but not prevent, levcromakalim-induced headache in healthy volunteers (Al-Karagholi et al., 2020). Oral glibenclamide also does not prevent CGRP-induced headache nor PACAP-38-induced headache in otherwise healthy patients (Coskun et al., 2021; Kokoti et al., 2022). This therapeutic failure of glibenclamide has been hypothesised to be due to poor receptor occupancy of K_ir_6.1/SUR2B K_ATP_ channel subtypes, thus development of novel selective K_ir_6.1/SUR2B channel inhibitors with good bioavailability is needed (Christophersen and Dyhring, 2023).

BKCa and Kv channels have also emerged as a potential candidates for migraine therapies. MaxiPost, a positive modulator of BKCa and neuronal K_v_7 channels, has been shown to induce migraine attacks in twenty-one (95%) patients with migraine without aura compared to none in the placebo arm (Al-Karagholi et al., 2021b). Additionally, retigabine, a K_v_7 channel opener, has been shown in animal studies to reduce transient receptor potential channels-induced CGRP release (Citak et al., 2022). Development of BKCa- and K_v_7-directed therapeutic drugs remains outstanding.

## 10 Glutamatergic pathway-targeted therapies

As the principal excitatory neurotransmitter within the central nervous system, glutamate has been extensively investigated as a potential therapeutic migraine target. Dysregulation of glutamatergic pathways may promote CSD and activation of the trigeminovascular system (Goadsby et al., 2017). Patients with chronic migraine have raised plasma glutamate concentrations and NR2B receptor expression (Tripathi et al., 2018). Moreover, CSF glutamate concentrations are significantly higher in CM patients with fibromyalgia than those without (Peres et al., 2004). Consequently, several glutamatergic receptor targets have been investigated, including ionotropic glutamate receptors such as N-methyl-D-aspartate (NMDA), α-amino-3-hydroxy-5-methyl-4-isoxazolepropionic acid (AMPA) and kainate receptors, as well as metabotropic glutamate receptors (mGluRs).

The evidence base for ketamine, a non-competitive NMDA receptor antagonist, is mixed. Intranasal administration of 25 mg ketamine doses have been shown in a double-blind trial to reduce the severity, but not duration, of aura in patients with migraine with prolonged aura (Afridi et al., 2013). However, intravenous 0.2 mg/kg ketamine infusions did not provide a statistically significant improvement in pain scores compared to placebo in patients presenting to an emergency department with acute migraine attacks (Etchison et al., 2018). However, dissociative adverse effects were common, including fatigue, nausea, and generalised discomfort (Etchison et al., 2018). Memantine, a low-affinity NMDA receptor antagonist, has been shown to be much better tolerated with oral memantine 10 mg once daily prophylaxis significantly reducing monthly migraine frequency, patient disability, and rescue medication use (Noruzzadeh et al., 2016; Shanmugam et al., 2019).

Tezampanel (LY293558), a non-competitive AMPA/kainate receptor antagonist, has demonstrated encouraging early clinical efficacy with 1.2 mg/kg intravenous infusions showing 2-h headache score improvement in 69% of patients compared to 86% and 25% of patients who received 6 mg subcutaneous sumatriptan and placebo interventions respectively (Sang et al., 2004). A related compound, LY466195 a selective kainate GluK1 receptor antagonist has been shown to attenuate trigeminovascular activation in rats (Andreou et al., 2015). However, Contrastingly, LY466195 has been reported to not affect vasodilatory responses induced in rats by endogenous nor exogenous CGRP administration (Chan et al., 2010). Ketamine and GYKI52466, an AMPA receptor antagonist, both attenuated CGRP-induced vasodilatation, while Ketamine and MK801, another NMDA receptor antagonist, both attenuated capsaicin- and electrically induced vasodilation (Chan et al., 2010). Translation of these findings to human subjects is currently outstanding; however, a single oral 250 mg dose of selurampanel (BGG492), another AMPA/kainate receptor antagonist, has been shown to induce 2-h pain freedom in 25% of patients experiencing acute migraine attacks, compared to 24% and 16% of patients who received 100 mg oral sumatriptan and a placebo control respectively (Gomez-Mancilla et al., 2014). BGG492, however, was inferior to sumatriptan in improving pain severity (54% versus 92%) and was also associated with a higher frequency of adverse effects (80% versus 56%) (Gomez-Mancilla et al., 2014).

Research into mGluRs as potential migraine targets has led to the development of several “glurants,” a class of receptor-modulating compounds. Raseglurant (ADX10059), an mGluR5 negative allosteric modulator, has been reported to attenuate dural vasodilator responses to meningeal stimulation in rats in a dose-dependent manner (Waung et al., 2016). These effects were comparable to that of naratriptan, while MK801 had no observable effect (Waung et al., 2016). Additionally, oral 375 mg single-doses of raseglurant induced 2-h pain freedom in 17% of human participants compared to 5% in the control arm (Waung et al., 2016). Though promising, interest in raseglurant has since diminished after another migraine study (NCT0082015) was terminated following reports that 120 mg twice-daily administration evoked significant hepatotoxicity (alanine transaminase levels greater than five times the upper limit of normal) in 16 (6%) patients (Waung et al., 2016). mGluR5, however, remains a key target candidate for novel migraine therapies. Moreover, other mGlu5 negative allosteric modulators, such as mavoglurant (AFQ056) and dipraglurant (ADX48621), have not shared similar hepatotoxicity concerns (Rascol et al., 2014).

Overall, glutamatergic pathway-targeted therapies have not yet produced an approved migraine treatment. Current evidence supports selective glutamate receptor modulation in migraine rather than broad inhibition of glutamatergic signalling, with NMDA, AMPA/kainate, and mGlu5 offering promising therapeutic targets.

## 11 Nitric oxide-targeted therapies

NO is an endogenous gaseous signalling molecule generated from L-arginine by neuronal, endothelial, and inducible nitric oxide synthases (nNOS; eNOS; and iNOS). The molecule regulates vascular tone and nociceptive signalling principally through activation of soluble guanylyl cyclase and increased intracellular cyclic guanosine monophosphate (cGMP). In migraine, NO-cGMP signalling also interacts with CGRP, ATP-sensitive potassium channels and other ion-channel mechanisms, providing several routes through which it may increase trigeminovascular excitability (Moncada et al., 1991; Olesen, 2008).

The strongest human evidence comes from nitroglycerin (NTG), an NO donor widely used as an experimental migraine model. In healthy volunteers, intravenous NTG produces a rapid, dose-dependent headache during infusion (Iversen et al., 1989). In a double-blind, placebo-controlled crossover study of migraineurs, NTG also produced a delayed headache peaking approximately 5.5 h after infusion; 8 of 10 participants completing the study developed attacks fulfilling criteria for migraine without aura, compared with one participant who received a placebo control (Thomsen et al., 1994). Migraineurs also demonstrate greater intracranial arterial sensitivity to NTG than healthy controls, supporting increased responsiveness to the downstream NO cascade rather than exposure to NO alone (Thomsen et al., 1993). Interestingly, an observational microbiome study identified higher abundances of nitrate-, nitrite- and NO-reducing oral bacteria among individuals reporting migraine (Gonzalez et al., 2016).

The therapeutic potential of NOS inhibition depends on the isoform targeted. eNOS is highly expressed within the vascular endothelium and is essential for maintaining normal vascular tone and cardiovascular homeostasis; systemic inhibition is therefore unlikely to be clinically tolerated. nNOS, by contrast, is distributed throughout the trigeminovascular system and central pain-processing pathways, including the trigeminal nucleus caudalis, periaqueductal grey, hypothalamus, thalamus and cerebral cortex (Ramachandran et al., 2010). In experimental models, NTG increases CGRP- and nNOS-immunoreactive neuronal expression within the trigeminal ganglion (Dieterle et al., 2011), while repeated triptan exposure increases nNOS expression in trigeminal dural afferents and enhances sensitivity to migraine triggers (De Felice et al., 2010). These findings provide a rationale for selectively inhibiting nNOS while preserving endothelial NO signalling.

In a small double-blind study of spontaneous migraine without aura, the non-selective NOS inhibitor L-NG-methylarginine produced 2-h headache relief in 10 (67%) treated participants, compared with two (14%) placebo-treated participants (Lassen et al., 1998). However, most placebo data were drawn from earlier trials, and the compound’s non-selective vascular effects restrict its therapeutic potential. Subsequent research into selective iNOS inhibitors, GW273629 and GW274150, have failed to reproduce these findings. In an open-label study of 15 migraineurs, GW273629 produced no improvement in headache nor associated symptoms; early drug exposure was 41% lower during attacks than interictally, which may have contributed to this negative result (Van der Schueren et al., 2009). A large two-part randomised, double-blind preventive trial evaluated GW274150 with 426 participants enrolled overall. Twelve-week courses of GW274150 at 60 nor 120 mg daily were no more effective than placebo for the primary endpoint nor any secondary endpoint, despite the given doses being predicted to inhibit iNOS by more than 80% (Høivik et al., 2010). These concordant negative studies suggest that iNOS is unlikely to be the principal source of therapeutically relevant NO signalling in migraine.

Another drug, NXN-188, was developed to combine nNOS inhibition with 5-HT_1B/1D_ receptor agonism. In a small randomised, double-blind crossover study in which treatment was administered during migraine aura, headache freedom at 2 hours occurred in 22% after NXN-188 and in 11% after placebo; however, the difference was not statistically significant, and only 18 participants completed both treatment periods (Hougaard et al., 2013). Its dual pharmacology also prevents any clinical effect from being attributed specifically to nNOS inhibition. Thus, genuinely selective nNOS inhibition remains biologically plausible but clinically unproven.

NO has also been investigated in relation to CSD. Though CSD can stimulate local NO release, experimental NOS inhibition has little effect on the accompanying regional cerebral blood-flow changes, suggesting that NO is not essential for CSD-related vascular responses (Read and Parsons, 1998; Zhang et al., 1994). Tonabersat is a benzopyran-derived gap-junction modulator developed to inhibit CSD and neuronal-glial communication rather than NO directly. It became relevant to NO-targeted migraine research because preclinical studies showed that it reduced both experimentally induced CSD and associated NO release (Read et al., 2000; Smith et al., 2000). Tonabersat has been shown to be ineffective as an acute migraine treatment (Dahlöf et al., 2009); however, in a preventive crossover trial tonabersat was reported to reduce the median number of aura attacks without significantly reducing overall migraine headache days (Hauge et al., 2009). This pattern may suggest that the drug’s clinical effects are mediated through CSD inhibition rather than direct suppression of NO signalling. Overall, the NO-cGMP pathway is strongly implicated in migraine generation, but successful therapeutic development will likely require selective modulation of nNOS or downstream signalling while preserving essential endothelial NO function.

## 12 Phosphodiesterase- and cyclic nucleotide-targeted therapies

Phosphodiesterases (PDEs) are enzymes that terminate intracellular signalling by hydrolysing the second messengers cAMP and cGMP, whose roles in migraine have been described in earlier sections of this review. PDE3 and PDE5 are expressed and enzymatically active within the rat trigeminal ganglion, where subsets of PDE-positive neurones co-localise with CGRP, supporting their involvement in trigeminovascular signalling (Nordgaard et al., 2014). Human provocation studies further demonstrate that pharmacological inhibition of PDE3 or PDE5, and the resulting accumulation of cAMP or cGMP respectively, can induce migraine-like headache (Guo et al., 2014; Kruuse et al., 2003; Al-Khazali et al., 2024; Al-Khazali et al., 2026b).

In a randomised, double-blind, placebo-controlled crossover study of participants with migraine without aura, the PDE3 inhibitor cilostazol induced delayed migraine-like attacks in 12 (86%) participants, compared with two (14%) following placebo, with a median onset of 6 h (Guo et al., 2014). Similarly, sildenafil, a selective PDE5 inhibitor, induced migraine in 10 (83%) participants, compared with two (17%) following placebo (Kruuse et al., 2003). The absence of significant changes in middle cerebral artery haemodynamics suggested that sildenafil-induced migraine could not be attributed solely to cerebral vasodilatation. Interestingly, sildenafil has been shown to exhibit sex-dependent effects, inducing headache but not migraine attacks in male patients with migraine without aura, while inducing both headache and migraine attacks in female patients (Mohammad et al., 2026). More recent randomised, double-blind, placebo-controlled crossover studies have extended these findings to persistent post-traumatic headaches (PPTH). Cilostazol induced migraine-like headache in 14 of 21 participants with PPTH, compared with three following placebo, while sildenafil induced attacks in 15 of 21 participants with PPTH, compared with four following placebo (Al-Khazali et al., 2024; Al-Khazali et al., 2026b). These findings implicate both cAMP- and cGMP-dependent signalling across clinically distinct headache phenotypes.

Furthermore, cyclic nucleotide signalling intersects with the CGRP pathway. CGRP-receptor activation stimulates adenylyl cyclase and increases intracellular cAMP; however, cilostazol provoked migraine at similar rates following erenumab or placebo pretreatment, suggesting that direct cAMP elevation can bypass upstream CGRP-receptor blockade (Do et al., 2023). Similarly, sildenafil was found to induce migraine attacks in 63% of erenumab-treated participants, compared with 19% who received placebo, indicating that cGMP-mediated migraine induction may occur independently of CGRP-receptor activation (Raffa et al., 2024).

Collectively, these findings identify cyclic nucleotide metabolism as a potential therapeutic target. Selectively enhancing PDE activity could theoretically accelerate cAMP or cGMP degradation and attenuate downstream trigeminovascular signalling. PDE activators have not yet been evaluated clinically in migraine; however, the widespread cardiovascular, vascular and platelet functions of PDE3 and PDE5 may limit safety and usability. Tissue-selective PDE enhancement or targeting downstream cyclic nucleotide effectors may therefore represent more feasible future strategies.

## 13 Conclusion

CGRP-targeted therapies have paved the way for novel pharmacological migraine treatments. CGRP-antagonists (gepants) and monoclonal antibodies have proven effective as both acute treatments for migraine and preventative therapies. Other emerging pathways remain in early, preclinical stages, highlighting gap between pathophysiological plausibility and robust clinical translation. PACAP ligand-directed monoclonal antibodies offer therapeutic promise in patients with treatment-resistant migraine. Despite robust preclinical experimental evidence foundations, no Amylin-targeted therapies have thus far been developed. The clinical failure of neurokinin-targeted therapies further challenges the hypothesis that substance P-mediated neurogenic inflammation represents a major pathogenic mechanism in human migraine. Ditans offer an alternative serotonergic therapeutic approach to established triptan therapies through selective 5-HT_1F_ receptor antagonism. This reflects a wider shift toward more selective receptor-based approaches. Further promising molecular targets include K_ir_6.1/SUR2B K_ATP_ and K_v_7 potassium channels, alongside GluK1 and mGluR5 glutamate receptors. Despite compelling mechanistic rationale, targeting orexinergic pathways, nitric oxide synthase, and phosphodiesterase/cyclic nucleotide signalling has thus far yielded disappointing results in the clinical development of effective migraine therapies. Overall, these emerging drug targets represent a shift toward mechanism-based migraine therapeutics; however, further translational research and larger-scale clinical trials are needed to establish efficacy and safety profiles.
